# Utility of Blood-Based Tau Biomarkers for Mild Cognitive Impairment and Alzheimer’s Disease: Systematic Review and Meta-Analysis

**DOI:** 10.3390/cells12081184

**Published:** 2023-04-18

**Authors:** Alex Zabala-Findlay, Lewis K. Penny, Richard A. Lofthouse, Andrew J. Porter, Soumya Palliyil, Charles R. Harrington, Claude M. Wischik, Mohammad Arastoo

**Affiliations:** 1 Institute of Medical Sciences, University of Aberdeen, Aberdeen AB25 2ZD, UK; 2 Scottish Biologics Facility, University of Aberdeen, Aberdeen AB25 2ZP, UK; 3 GT Diagnostics, Aberdeen AB24 5RP, UK; 4 TauRx Therapeutics Ltd., Aberdeen AB24 5RP, UK

**Keywords:** Alzheimer’s disease, mild cognitive impairment, tau, biomarkers, diagnostics, blood

## Abstract

Objectives: With the development of new technologies capable of detecting low concentrations of Alzheimer’s disease (AD) relevant biomarkers, the idea of a blood-based diagnosis of AD is nearing reality. This study aims to consider the evidence of total and phosphorylated tau as blood-based biomarkers for mild cognitive impairment (MCI) and AD when compared to healthy controls. Methods: Studies published between 1 January 2012 and 1 May 2021 (Embase and MEDLINE databases) measuring plasma/serum levels of tau in AD, MCI, and control cohorts were screened for eligibility, including quality and bias assessment via a modified QUADAS. The meta-analyses comprised 48 studies assessing total tau (t-tau), tau phosphorylated at threonine 181 (p-tau181), and tau phosphorylated at threonine 217 (p-tau217), comparing the ratio of biomarker concentrations in MCI, AD, and cognitively unimpaired (CU) controls. Results: Plasma/serum p-tau181 (mean effect size, 95% CI, 2.02 (1.76–2.27)) and t-tau (mean effect size, 95% CI, 1.77 (1.49–2.04)) were elevated in AD study participants compared to controls. Plasma/serum p-tau181 (mean effect size, 95% CI, 1.34 (1.20–1.49)) and t-tau (mean effect size, 95% CI, 1.47 (1.26–1.67)) were also elevated with moderate effect size in MCI study participants compared to controls. p-tau217 was also assessed, albeit in a small number of eligible studies, for AD vs. CU (mean effect size, 95% CI, 1.89 (1.86–1.92)) and for MCI vs. CU groups (mean effect size, 95% CI, 4.16 (3.61–4.71)). Conclusions: This paper highlights the growing evidence that blood-based tau biomarkers have early diagnostic utility for Alzheimer’s disease. Registration: PROSPERO No. CRD42020209482.

## 1. Introduction

Alzheimer’s disease (AD) is a debilitating neurodegenerative disease associated with progressive cognitive decline. AD is the most common type of dementia and a major global burden. Approximately 50 million people were reported to be suffering from dementia in 2018, and this number is expected to triple by 2050 [1]. Due to an ever-increasing elderly population, dementia now kills more people in the U.S. than prostate cancer and breast cancer combined and is the leading cause of death in both England and Wales [1].

Quantification of the amounts of total tau (t-tau) and phosphorylated tau (p-tau) have been found to correlate with loss of cognition and with neurodegeneration in autopsied AD patients [2,3] and in antemortem CSF [4]. For clarity, t-tau is quantified using antibodies to an epitope in the mid region of tau that recognizes all six isoforms of tau in the human brain. Several studies have demonstrated that some mid-domain and C-terminal p-tau residues are elevated in AD [5]. However, the most investigated to date has been tau phosphorylated at threonine 181 (p-tau181) [6]. A previous systematic review showed that t-tau and p-tau were elevated by 2.54 and 1.88-fold, respectively, when comparing AD CSF to control CSF [7]. In addition to positron emission tomography (PET) imaging, t-tau and p-tau CSF biomarkers are currently being utilized to support the clinical diagnosis of patients on the AD continuum and to identify individuals in the preclinical stages of the disease [8,9,10].

Given that the presymptomatic and prodromal stages of the AD continuum are extensive, it is important to diagnose AD as early as possible in order to identify the extent of disease progression and increase the chance of treating patients with stratified medicines before the advanced progression of the disease renders preventative treatment ineffective [10]. Thus, biomarker-based diagnosis at the earliest opportunity is an important goal for AD. The first detectable changes in CSF biomarkers of t-tau and p-tau arise 17 and 21 years before the symptomatic onset of AD, respectively, truly highlighting their utility as early AD diagnostics [11].

However, CSF sampling involves potentially dangerous and certainly unpleasant lumbar punctures, while PET imaging is limited to only the best-equipped medical facilities. Considering the global burden of AD, CSF sampling and PET imaging are not ideal applications to deploy in population-wide screening for early AD diagnostics. For example, it was estimated in 2015 that around 480 million people worldwide were at either Braak stage 2 (early stages of AD/tau pathology) or at more advanced stages [12]. Recent advances in ultrasensitive technologies and novel approaches now allow tau-based biomarkers to be assessed in the peripheral matrix of blood. These approaches include single molecule array (SIMOA) immunomagnetic reduction (IMR) and Meso Scale Discovery (MSD) platforms, as well as next-generation mass spectrometry [13,14,15,16]. There has been a plethora of studies on blood-based tau biomarkers over the last decade. This timely systematic review aims to assess the ratio of biomarker concentrations for t-tau and p-tau in MCI and AD in comparison to cognitively unimpaired (CU) controls.

## 2. Methods

### 2.1. Search Strategy

Embase and MEDLINE were searched for primary studies published from 1 January 2012 to 1 May 2021 assessing tau plasma/serum biomarkers in AD patients with a control cohort. This primary search included (Alzheimer) AND (plasma OR serum OR blood) AND (biomarker OR biological marker) AND (tau OR t-tau OR p-tau OR ttau OR ptau) keywords in the title or abstract. Studies were limited to human subjects, publications in the English language, and ‘article’ as publication type to limit conference proceedings, systematic reviews, or reviews. To reduce the number of studies for screening, titles containing words indicating a review or systematic review were excluded (Table 1). Studies conducted in Down syndrome populations were excluded. This systematic review was registered on the International Prospective Register of Systematic Reviews (PROSPERO) on 13 October 2020 (PROSPERO Registration No. CRD42020209482). Additionally, further studies were identified through cross-referencing by identifying citations in eligible studies. Study identification and reduction were conducted and independently verified by the lead authors.

Studies were excluded if they did not present plasma or serum tau (t-tau or p-tau) concentrations, in mean ± standard deviation (SD) or standard error mean (SEM), or if there was no data for either AD or MCI compared to a control group. Furthermore, studies were required to specify the diagnostic criteria used to group study participants into CU, AD, and/or MCI and follow published criteria.

### 2.2. Data Extraction

Data were extracted for the CU cohort from those closest in age to the MCI and AD groups if more than one CU cohort was reported. Additionally, if study participants were grouped by Aβ status, CU participants were those who were Aβ-, and MCI participants were Aβ + (in accordance with NIA-AA diagnostic guidelines) [17]. If the same cohort was studied in more than one study and the inclusion criteria were met, the study with the lower number of participants was excluded as a duplicate. 

If all criteria were met but there was no plasma/serum tau concentration reported in mean ± SD or mean ± SEM, authors were contacted and asked to supply this information. Data for t-tau and p-tau plasma or serum concentrations were extracted from cross-sectional studies or at baseline in longitudinal studies. A quality assessment was carried out using a checklist that has been used in a previous systematic review for AD diagnostic biomarkers [7] (Supplementary Materials Table S3), with elements from the Quality Assessment of Diagnostic Accuracy Studies (QUADAS) tool, Standards for Reporting Diagnostic Accuracy (STARD), and A Measurement Tool to Assess Systematic Reviews (AMSTAR) [18,19,20].

### 2.3. Data Analysis

Plasma and serum analyses for each biomarker were meta-analyzed together. Meta-analyses were carried out based on the ratio of the means (RoM) of the disease cohort divided by the control cohort (i.e., AD/CU or MCI/CU) in order to determine the effect size. This method is useful for comparing biomarker concentrations obtained from different assays, which can vary substantially between laboratories and assays [21]. If the resulting ratio is above 1, the biomarker concentrations are greater in the disease cohort than in the control cohort, and if the ratio is below 1, the levels in the control cohort are higher. This method has been used in a previous AD biomarker systematic review [7] and was carried out as described by [22]. The 95% confidence interval (CI) was calculated for each ratio following the delta method. The overall effect and its 95% CI followed random-effects meta-analysis methods, as described previously [22,23] (Supplementary Figure S1B). Finally, the weight of each study was determined by the inverse of the variance to help identify studies with stronger results, i.e., larger sample size and/or smaller variance (Supplementary Figure S1B). 

Meta-analysis forest plots were created using Excel 16.0. For studies with more than one study arm (e.g., derivation and validation cohorts), each arm was treated as a separate study but labeled accordingly. Tests for heterogeneity between studies involved the chi-squared statistic, Q [22], which was used to calculate I^2^ (where *I*^2^ = 100% × (*Q* − df)/*Q*; where df is degrees of freedom), a measure of inconsistency which represents the percentage of the chi-squared statistic not explained by the variance within the studies [24]. Due to high heterogeneity and in an attempt to limit this, sub-group analysis was also performed as per above for each technology.

## 3. Results

### 3.1. Study Inclusion 

**Figure 1:**
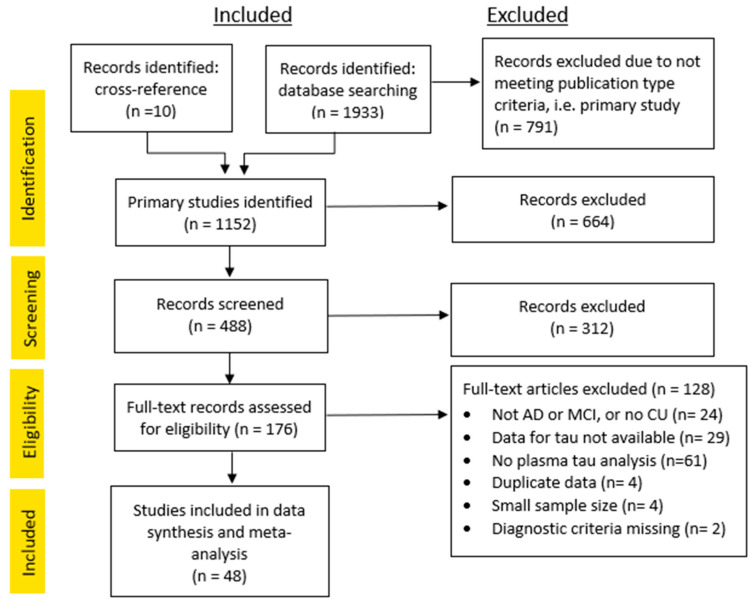
Study inclusion flow diagram.

The database search returned 1933 results, and a further 10 studies were identified through cross-referencing. After excluding studies that did not match the study type (i.e., systematic reviews, conference proceedings, etc.), did not involve human subjects, were not published in English, or were published prior to 2012, 488 studies remained. The abstracts for these 488 studies were screened against the criteria described in Methods. From this, 312 studies were excluded, and 176 remained for full-text assessment for eligibility. A further 128 studies were excluded for various reasons, as indicated in the flow diagram (Figure 1). This left 48 studies eligible for inclusion in data synthesis for this meta-analysis. 

Of the 48 included studies, there were data on both plasma/serum t-tau and p-tau181 in 11 studies, a total of 21 cohorts from which p-tau181 concentrations were collected, and a total of 42 cohorts collecting t-tau concentrations. A total of 41 studies reported data for t-tau in AD patients vs. CU, 25 studies reported data on t-tau in MCI vs. CU, 20 studies included data on p-tau181 in AD vs. CU, 12 studies included data on p-tau181 in MCI vs. CU, 3 studies included p-tau217 data in AD vs. CU, 2 included p-tau217 in MCI vs. CU, and 1 included p-tau231 in AD vs. CU as well as MCI vs. CU.

### 3.2. Systematic Review and Quality Assessment

Data from a total of 15,713 study participants: CU = 10,340, MCI = 2363, AD = 2880 were collected from the 48 included studies. The studies included in the data synthesis were mainly longitudinal, where blood samples were taken at baseline, and cross-sectional studies, where patient groups were compared at an individual time point. 

Data were reported in mean (SD) pg/mL for all the cohorts except for 6 cohorts from 3 different studies, where data were reported in median (IQR) pg/mL or arbitrary units (AU). For t-tau, the concentrations reported in all studies ranged from a mean (SD) of 0.32 (0.32) pg/mL to 530 (193.6) pg/mL in the AD group, from 0.31 (0.16) pg/mL to 729.8 (225.6) pg/mL in the MCI group, and from 1.6 (0.9) pg/mL to 819.5 (294.4) pg/mL in the CU group. For p-tau181, the concentrations reported ranged from a mean of 0.17 (0.17) pg/mL to 150 (57.7) pg/mL in the AD group, from 2.9 (1) to 22.8 (9.9) in the MCI group, and from 0.04 (0.08) pg/mL to 107 (57.8) pg/mL in the CU group. For p-tau217, the concentrations reported ranged from a mean of 0.32 (0.17) pg/mL to 7.75 (5.34) pg/mL in the AD group, from 0.31 (0.16) to 4.56 (3.11) in the MCI group, and from 0.07 (0.03) pg/mL to 1.79 (1.95) pg/mL in the CU group. In the only eligible study for ptau231, reported concentrations were 29.22 (8.20) pg/mL in the AD group, 19.45 (7.10) pg/mL in the MCI group, and 14.94 (4.10) pg/mL in the CU group. The biomarker data extracted from the studies, as well as the assays used in each study, have been summarised in the Supplementary Materials (Table S1). The studies used a variety of assay formats, but the type of assay could be broadly divided into 5 categories: Single Molecule Array (SIMOA: 14 studies t-tau, 14 studies p-tau181, 2 studies p-tau217, 1 study p-tau231), immunomagnetic reduction (IMR: 15 studies t-tau, 4 studies p-tau181), electrochemiluminescence (ECL: inclusive of Elecsys-ECL and MSD-ECL: 3 studies t-tau, 2 studies p-tau217), enzyme-linked immunosorbent assay (ELISA: 9 studies t-tau, 2 studies p-tau181), and liquid chromatography–mass spectrometry (LC–MS: 1 study t-tau, 1 study p-tau181, 1 study p-tau217). There was a total of 30 distinct assays from all cohorts. Data for all cohorts were obtained using commercial assays except in 9 studies, 6 of which employed ELISA and 3 which employed “homebrew” SIMOA. Despite using commercial assays, several studies implemented variations to the quantification of plasma/serum biomarkers, such as using antibodies outside the commercial kit or calibrating a commercial assay with antibodies from a different commercial assay. A table summarising details for all included studies is presented in the supplementary Appendix (Table S2).

The most common diagnostic criteria used in studies were those developed by the NIA-AA [25]. Others included the Diagnostic and Statistical Manual of Mental Disorders (DSM-IV) [26], the International Classification of Diseases (ICD10), Petersen criteria for MCI [27], and the National Institute of Neurological and Communicative Diseases and Stroke/Alzheimer’s Disease and Related Disorders Association criteria (NINCDS-ADRDA) [28]. The quality of all studies was assessed as high with low bias using the modified QUADAS, and this is presented in the Supplementary Materials (Table S4).

### 3.3. Meta-Analysis

The included studies were split into groups to carry out the meta-analyses: t-tau in AD vs. CU (n = 39), p-tau181 in AD vs. CU (n = 20), p-tau217 in AD vs. CU (n = 3), t-tau in MCI vs. CU (n = 24), p-tau181 in MCI vs. CU (n = 13), p-tau217 in MCI vs. CU (n = 2). Summary statistics presented as median and interquartile range prevented pooling of the data, and subsequently, 5 cohorts [29,30,31] were not included in these meta-analyses. 

#### 3.3.1. p-tau181

**Figure 2:**
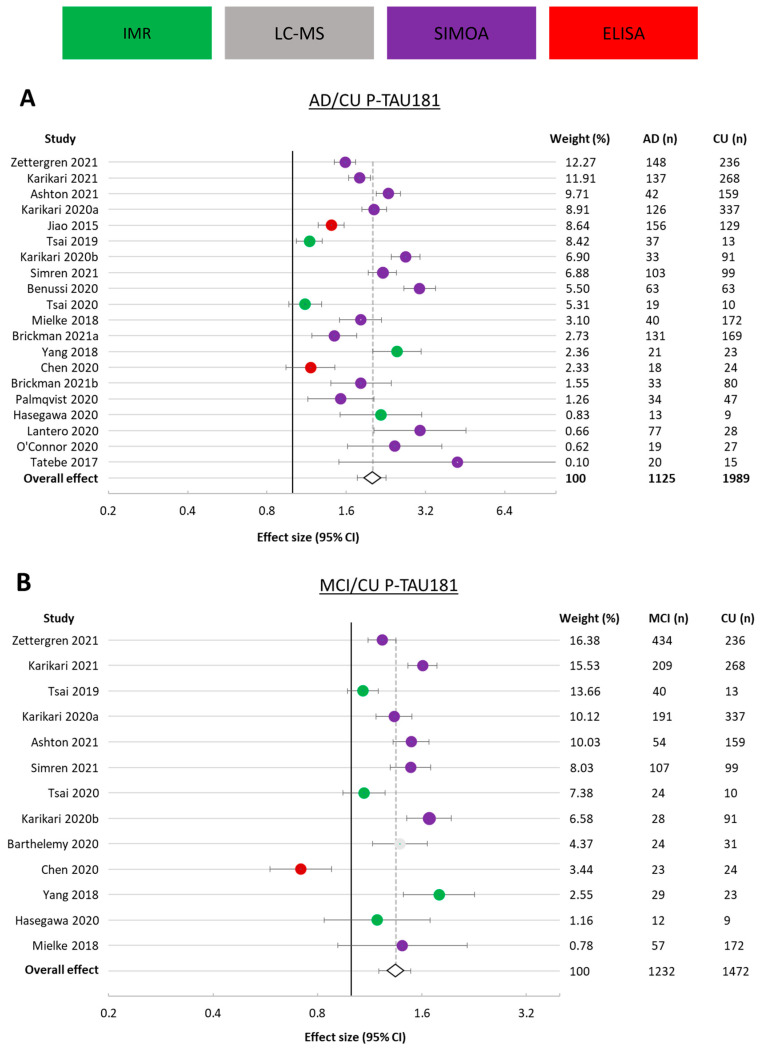
Forest plots for studies presenting plasma/serum p-tau181. (**A**). Alzheimer’s disease (AD) versus cognitively unimpaired (CU) participants. (**B**). Mild cognitive impairment (MCI) versus cognitively unimpaired (CU) participants. Ratio (effect size) and 95% CI for individual studies are presented by a circle, color-coded by assay type; the overall ratio is presented by a diamond and a grey vertical line. Studies are arranged in descending weight order. Data are presented on a log scale. An effect size of 1 indicates equal biomarker concentrations in AD and CU. Percentage weight, number of AD participants, and number of CU participants are displayed on the right side of the forest plot for each study and the combination of all studies. AD: Alzheimer’s disease; CU: cognitively unimpaired; CI: confidence interval.

From the studies eligible for meta-analysis that presented data on plasma/serum p-tau181 concentrations, a total of 1233 AD and 2017 CU study participants were included in the meta-analysis for assessing the ratio of p-tau181 in AD and control participants (Figure 2A). Of these studies, 8 did not include an MCI cohort, making a total of 1169 MCI and 1459 CU participants involved in determining the ratio of p-tau181 in MCI to CU participants (Figure 2B). Except for one study reporting an effect size of 0.72, all cohorts in these comparisons had an effect size greater than 1, indicating higher p-tau181 concentrations in AD and MCI than in CU participants. The average ratio (effect size) of AD to CU was 2.02 (95% CI 1.76–2.27), and 1.34 (95% CI 1.20–1.49) for MCI to CU.

From these 34 studies, data were obtained using 5 different assay formats (IMR, SIMOA, ELISA, ECL, and LC-MS). In the meta-analysis for AD versus CU, the greatest weighting contribution to the overall effect was SIMOA (72.1%), followed by IMR (25.56%) and ELISA (2.34%). In the meta-analysis for MCI versus CU, the greatest weighting contribution was again from SIMOA (67.45%), followed by IMR (24.74%), LC-MS (4.37%), and ELISA (3.44%). The spread of results across studies was large, with confidence intervals from several studies not overlapping. Individual study variance was different across studies, as indicated by their weight (i.e., inverse of variance). The between-study heterogeneity was considerable for both AD versus CU (I^2^ = 98.23%) and for MCI versus CU (I^2^ = 92.17%).

#### 3.3.2. t-tau

**Figure 3:**
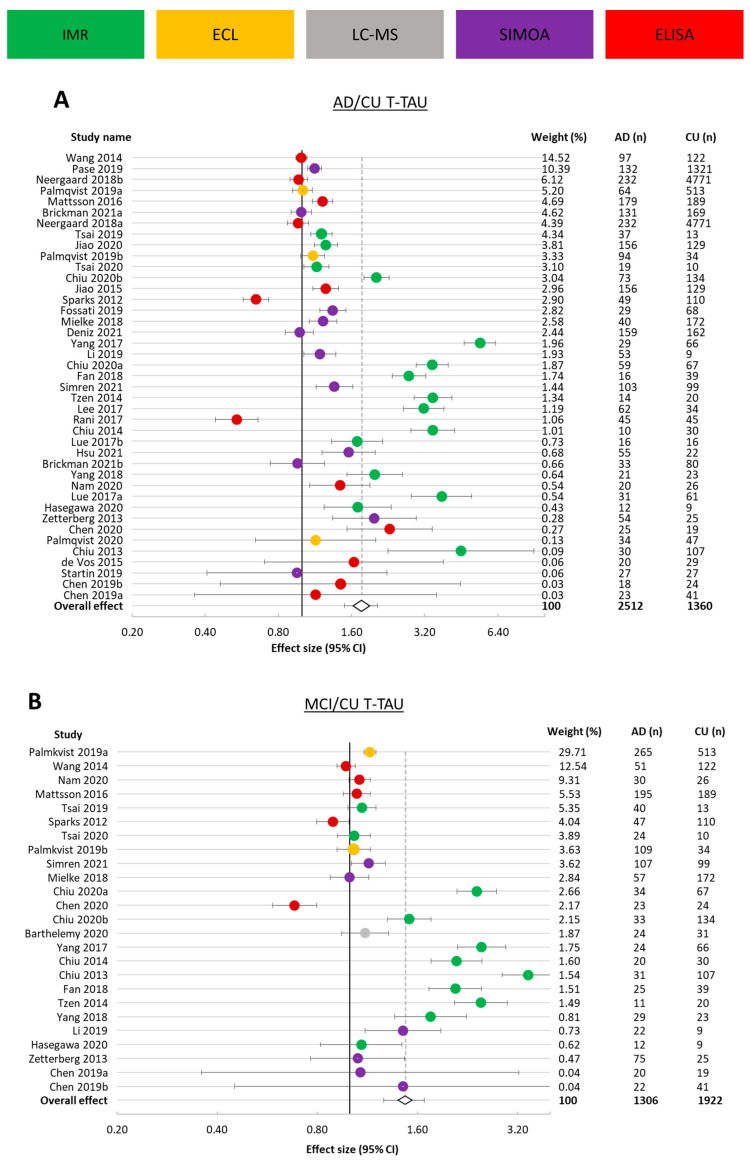
Forest plot for studies presenting plasma/serum t-tau. (**A**). Alzheimer’s disease (AD) versus cognitively unimpaired (CU) participants. (**B**). Mild cognitive impairment (MCI) versus cognitively unimpaired (CU) participants. Ratio (effect size) and 95% CI for individual studies are presented by a circle, color-coded by assay type, and the overall ratio is presented by a diamond and a grey vertical line. Studies are arranged in descending weight order. Data are presented on a log scale. An effect size of 1 indicates equal biomarker concentrations in AD and CU. Percentage weight, number of AD participants, and number of CU participants are displayed on the right side of the forest plot for each study and the combination of all studies. AD: Alzheimer’s disease; CU: cognitively unimpaired; CI: confidence interval.

A larger number of studies were available for t-tau biomarkers than for p-tau181, with 42 studies being eligible for inclusion in the meta-analysis. The total number of study participants in these cohorts was 2359 AD patients, 1373 MCI patients, and 8994 CU individuals. As one study did not investigate t-tau in AD patients, 8970 CU individuals were pooled into the meta-analysis assessing plasma/serum t-tau ratio in AD to CU (Figure 3A), and as 17 studies were not eligible for the meta-analysis assessing the ratio in MCI to CU participants, 1974 CU study participants were included in this analysis (Figure 3B).

The average ratio (effect size) was 1.77 (95% CI 1.49–2.04) in AD to CU and 1.47 (95% CI 1.26–1.67) in MCI to CU. Of the 41 studies assessing t-tau in AD versus CU, 9 had a ratio of 1 or below, with a minimum effect size of 0.68 and a maximum of 4.52. The spread of results was wide, with an I^2^ value of 99.36%. Similarly, the MCI to CU ratio was 1 or below in 4 of the 24 studies, and the I^2^ value was 98.6%.

These results were obtained using 5 different assay formats, where studies in the AD versus CU meta-analysis that used ECL and IMR carried less weight overall (8.54% and 25.84%) than those using SIMOA and ELISA (28.10% and 37.52%). For MCI versus CU, studies using ELISA carried the most weight (33.58%), followed by electrochemiluminescence (33.43%), IMR (23.37%), SIMOA (7.74%), and lastly, LC-MS (1.87%).

#### 3.3.3. p-tau217

**Figure 4:**
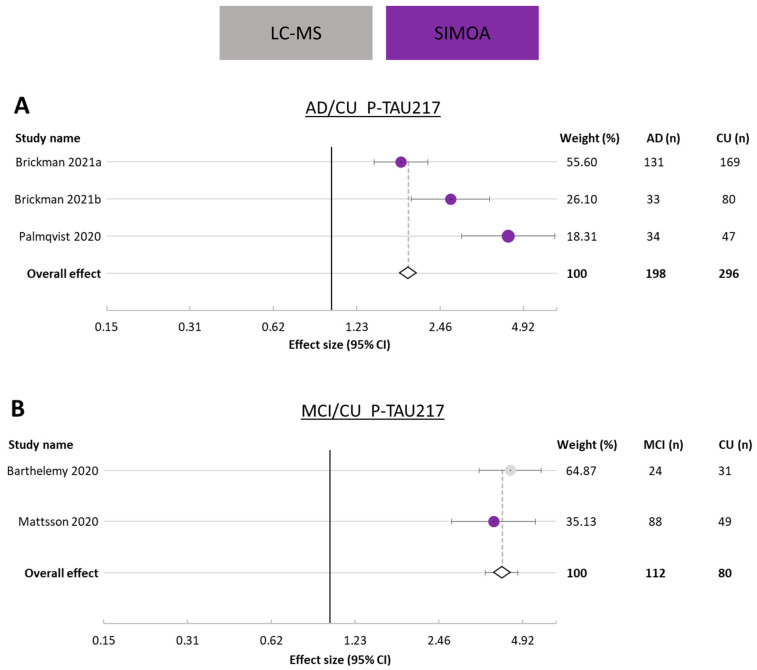
Forest plot for studies presenting plasma p-tau217. (**A**). Alzheimer’s disease (AD) versus cognitively unimpaired (CU) participants. (**B**). Mild cognitive impairment (MCI) versus cognitively unimpaired (CU) participants. Ratio (effect size) and 95% CI for individual studies are presented by a circle, color-coded by assay type, and the overall ratio is presented by a diamond and a grey vertical line. Studies are arranged in descending weight order. Data are presented on a log scale. An effect size of 1 indicates equal biomarker concentrations in AD and CU. Percentage weight, number of AD participants, and number of CU participants are displayed on the right side of the forest plot for each study and the combination of all studies. AD: Alzheimer’s disease; CU: cognitively unimpaired; CI: confidence interval.

As p-tau217 is a more recently identified AD biomarker in plasma [29,32], very few p-tau217 studies were available for inclusion in the meta-analysis. Three studies were included for assessing the ratio of p-tau217 in AD vs. CU (Figure 4B), and two were included for assessing the ratio of p-tau217 in MCI vs. CU (Figure 4B). A total of 198 AD and 296 CU study participants were included in the AD vs. CU analysis, and a total of 73 MCI and 119 CU study participants were included in the MCI vs. CU analysis. All cohorts in these comparisons had an effect size greater than 1, indicating higher p-tau217 concentrations in AD and MCI groups than in CU participants. The average ratio (effect size) of AD to CU was 1.89 (95% CI 1.86–1.92), and 4.16 (95% CI 3.61–4.71) for MCI to CU, with I^2^ of 99.986% and 84.77%, respectively. For these studies, data were obtained using 2 different assay types (SIMOA and LC-MS).

### 3.4. Comparison of Ultrasensitive Technologies

Sub-analysis by technology platforms was performed for t-tau and ptau181, suggesting that immunomagnetic reduction (IMR) and single molecule array (SIMOA) approaches show a greater difference in these biomarkers between CU control individuals and participants within the AD continuum. When comparing AD patients to CU individuals, studies using SIMOA to assess p-tau181 had an overall effect size of 2.09 (95% CI 2.13–2.05), which was superior to IMR 1.38 (95% CI 1.46–1.30) and ELISA 1.35 (95% CI 1.45–1.25). In studies assessing t-tau, IMR had an average effect s^i^ze of 2.29 (95% CI 2.33–2.25), which was superior to SIMOA 1.24 (95% CI 1.56–0.92), ELISA 1.16 (95% CI 1.53–0.8), and ECL 1.05 (95% CI 1.12–0.97).

When comparing MCI patients to CU individuals, included studies only used SIMOA and IMR to assess p-tau181 with an average effect size of 1.46 (95% CI 1.84–1.08) and 1.28 (95% CI 1.79–0.78), respectively. In studies assessing t-tau, IMR had an overall effect size of 1.95 (95% CI 2.25–1.65), which was superior to SIMOA 1.11 (95% CI 1.19–1.03), ECL 1.09 (95% CI 1.79–0.39), and ELISA 0.99 (95% CI 1.02–0.95) (Supplementary Materials Table S3A,B).

This sub-analysis was also performed in an attempt to further combat the high heterogeneity seen in these overall meta-analyses; however, this proved unsuccessful, and the heterogeneity remained high (91–99%) even within the same technology used.

### 3.5. Overall Effects

**Figure 5:**
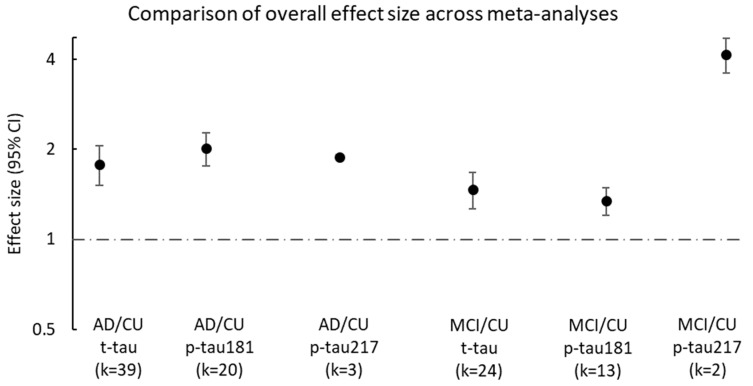
Overall effect size from each of the meta-analyses. The 6 meta-analyses are presented side by side and include plasma/serum p-tau181, p-tau217, and t-tau ratios of AD to CU and MCI to CU. Ratio (effect size) and 95% CI for each study are presented on the y-axis, where a ratio above 1 indicates greater biomarker concentrations in Alzheimer’s disease participants than in cognitively unimpaired participants. AD: Alzheimer’s disease; CU: cognitively unimpaired; MCI: mild cognitive impairment; k: number of studies pooled together in each meta-analysis; t-tau: total tau; p-tau181: phosphorylated tau at threonine 181; p-tau217: phosphorylated tau at threonine 217.

The overall effect sizes from the meta-analyses presented here are summarised in Figure 5. p-tau181 (average effect size: 2.02, 95% CI, 1.76–2.27, no. of studies = 20) and t-tau (average effect size: 1.77, 95% CI, 1.49–2.04, no. of studies = 39) were elevated when comparing the AD group to the CU group. p-tau181 (average effect size: 1.34, 95% CI, 1.20–1.49, no. of studies = 13) and t-tau (average effect size: 1.47, 95% CI, 1.26–1.67, no. of studies = 24) were also both elevated in the MCI group compared to the CU group, albeit the effect sizes were slightly lower than the AD vs. CU comparison. The limited studies on p-tau217 also suggest that this biomarker is discriminatory and higher to a similar degree when comparing AD to CU (average effect size: 1.89, 95% CI 2.05–2.13, no. of studies = 3). The highest effect size was noted with p-tau217 levels when comparing MCI to CU (average effect size: 4.16, 95% CI 3.61–4.71, no. of studies = 2).

## 4. Discussion

This systematic review set out to analyze current evidence on plasma/serum tau biomarkers and their ability to discriminate MCI and AD populations from CU. Through a systematic search of two databases, 48 appropriate studies were identified, which encompassed a total of 51 cohorts and 15,646 participants. Plasma/serum p-tau181, p-tau217, and t-tau were determined to be elevated in AD and MCI groups compared to the CU group, with positive effect sizes of AD to CU ratio for p-tau181 (2.02, 95% CI 1.76–2.27), p-tau217 (1.89, 95% CI 1.86–1.92), and t-tau (1.77, 95% CI 1.49–2.04), and positive effect sizes for ratio in MCI to CU for p-tau181 (1.34, 95% CI, 1.20–1.49), p-tau217 (4.16, 95% CI 3.61–4.71), and t-tau (1.47, 95% CI 1.26–1.67). These results provide evidence that p-tau181, p-tau217, and t-tau have potential diagnostic utility as plasma/serum biomarkers for the early stages of the AD continuum.

A previous systematic review, incorporating studies up to 2014, employed the same ratio-of-means method to analyze data from CSF samples [7]. The authors reported associations between AD and increased t-tau (average effect size: 2.54, 95% CI 2.44–2.64) and p-tau181 (average effect size: 1.88, 95% CI 1.79–1.97) when compared to controls. Despite only having limited studies available at the time (6 studies with 271 AD patients and 394 CU controls), the plasma/serum t-tau ratio was also obtained (average effect size: 1.95, 95% CI 1.12–3.38). p-tau181 was not assessed as there was not enough published literature at the time to carry out a meta-analysis. The results from this up-to-date systematic review are in line with those previously presented [7], with the addition that the findings are also evident in the prodromal stages of AD, as an MCI group was taken into consideration in the present study. Plasma p-tau181 has been shown to gradually increase along the AD continuum, with the lowest levels detected in amyloid β-negative young adults and cognitively unimpaired older adults, higher levels in amyloid β-positive cognitively unimpaired older adults and MCI patients, and highest levels in amyloid β-positive MCI and AD patients. p-tau181 has also been proven to be valuable in distinguishing AD patients from patients suffering from other tauopathies, dementias, or co-morbidities [33,34], which is not the case for t-tau [35].

The findings of this meta-analysis need to be considered alongside the heterogeneity noted between each study (I^2^ values of up to 99.36% [24]). Contributory factors for this are extensive and include but are not limited to different and evolving diagnostic criteria [8,9,10], subjective clinical assessment and diagnosis [36], clinical and pathological heterogeneity of AD itself [37], the inclusion of familial and sporadic cases, sample handling, processing, and pre-analytical variables [38], as well as different assays and technologies used to derive the data. The variability in quantification and measurement of potential AD biomarkers for CSF samples has been noted previously to arise in assays performed between and within laboratories and between commercial-research-use-only assays and assays developed in-house [21]. In an attempt to reduce heterogeneity, meta-analyses were carried out based on the ratio of the means (RoM) of the disease cohort divided by the control cohort (i.e., AD/CU or MCI/CU) rather than using traditional and quantifiable cut-off levels. The former method is useful to compare biomarker concentrations obtained using different assays, which vary substantially between laboratories and assays.

To further combat heterogeneity, a subsequent meta-analysis was performed for both t-tau and p-tau181 for each individual technology; however, this proved that heterogeneity remains high (91–99%) even where the same technology platform has been used. Through the harmonization of sample-to-analysis procedures, heterogeneity between future studies could be reduced. To this end, the Alzheimer’s Association has established the Global Biomarker Standardization Consortium (GBSC) to achieve consensus on best practices to validate and standardize biomarker tests for use in clinical practice. The GBSC has further set up several initiatives with the aim of reducing heterogeneity between studies and validating diagnostic tests on a global scale. These include a reference materials program established to develop universal reference material for fluid biomarkers, a reference methods program to develop ideal protocols, and the standardization of Alzheimer’s blood biomarkers (SABB) program, which was launched in 2018, with the aim of standardizing pre-analytical factors in blood sample collection, handling, and processing [39].

**Figure 6:**
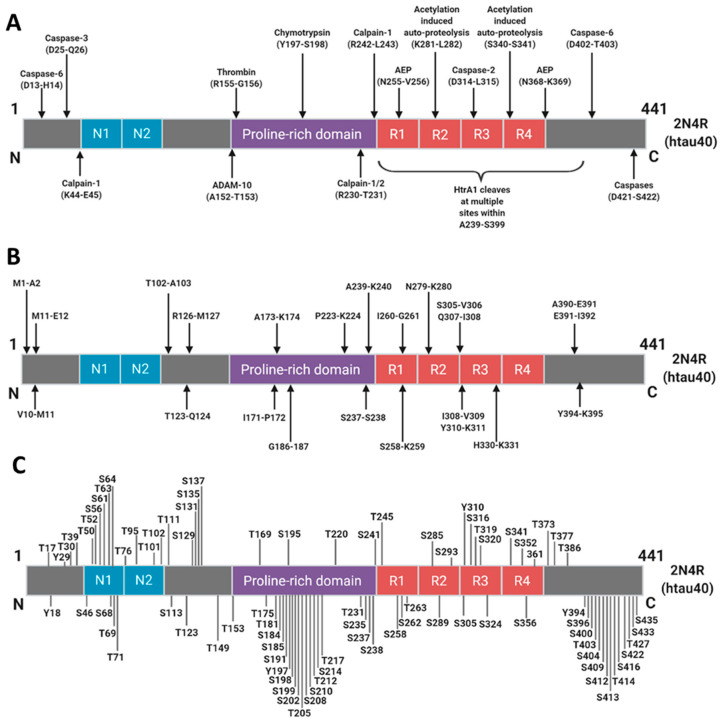
Sites for truncation and phosphorylation of human tau (2N4R): Proteolytic cleavage sites where known proteases are responsible (**A**) or where the protease responsible has yet to be identified (**B**). Putative tau phosphorylation sites (**C**). Those below each of the 2N4R tau schematics are sites that have been identified in the AD brain. Information derived from [46]. Reprinted from [47]. Copyright (2018) IOS Press and authors under terms of CC BY-NC 4.0.

Tau protein is fascinatingly complex in its physiology and capacity to cause disease, transitioning from a natively unfolded form to insoluble aggregates and paired helical filaments in AD. It is associated with a multitude of post-translational phosphorylation and truncation events (Figure 6A–C) [40]. With various truncated fragments in mind and the variation of antibodies used throughout tau biomarker studies, it is unlikely that most of these antibodies or antibody pairings are detecting ‘total’ full-length tau, but rather, each assay is detecting a different pool from a milieu of tau fragments. It may, therefore, be of great benefit if we move away from considering tau as a single biomarker but rather look deeper into the composition of different tau fragments within biological fluids. A measurement, therefore, based on the ratio of different tau fragments may also prove beneficial in reducing false negative/false positive results and overcoming inter-individual variation, as is the case when the Aβ42/40 ratio is measured in CSF [41,42]. It has also been shown that an antibody pairing that recognizes an N-terminal fragment was able to distinguish patients on the AD continuum from healthy control patients, whereas an antibody pairing detecting the full-length tau protein could not [43]. One common component that links all of the assays considered here, despite the different technology platforms and regions of tau protein detected, is a reliance on antibody-based recognition. It seems justified to speculate, therefore, that this antibody variation is likely to be a significant component of the heterogeneity revealed by this meta-analysis, and further studies are needed to explore antibody pairings and epitopes with improved diagnostic potential. It should be noted that this exploration of tau protein epitopes also applies to phospho-tau-based assays. It was recently shown that the detection of tau in CSF, using a combination of an N-terminal antibody paired with a p-tau181 capture antibody, improved diagnostic accuracy for the detection of prodromal AD when compared to antibodies directed toward the mid-region domain of tau [33]. Of course, p-tau181 is not the only phospho-tau biomarker. Tau can be phosphorylated at multiple sites (Figure 6C), and while there are surprisingly similar phosphorylation patterns between physiological and pathological tau, some of these sites are thought to be involved in the progression of AD [44]. This opens an opportunity to investigate other tau phosphorylation sites as potential AD diagnostic biomarkers. p-tau217 and p-tau231 have recently emerged as promising biomarker candidates [28,32,44]. Our meta-analysis on the limited number of eligible studies on p-tau217 suggests that it performs similarly to p-tau181 in the AD group vs. the CU group but outperforms p-tau181 in the MCI group vs. the CU group. A meta-analysis was not performed for p-tau231 as there was only one study eligible for inclusion. This study showed that p-tau231 could differentiate AD and MCI groups from a CU group with high accuracy in plasma samples [45].

## 5. Conclusions

Diagnosing Alzheimer’s disease at an early stage, and in particular, from a convenient blood sample, has been the focus of many studies over the past decade. Such a valuable test would provide significant benefits for patients and healthcare systems. These benefits would include: meeting the demands of the increasing numbers of people seeking a diagnosis; allowing precious time for patients to make lifestyle changes and manage their affairs; improving patient stratification into clinical trials; and permitting potential therapeutic intervention upon early diagnosis. Furthermore, identifying dynamic AD-specific blood biomarkers can serve as an important screening tool for clinical observation of patient response to treatment. The results, summarised in this systematic review, provide evidence for the utility of plasma/serum t-tau, p-tau181, and p-tau217 as diagnostic biomarkers for AD. It also suggests that these biomarkers may be able to predict the future development of AD because sensitive assays allow them to distinguish between CU individuals and MCI patients on the AD continuum. Such evidence signifies a promising future for AD blood-based diagnostics, and with the increasing number of studies currently being performed, as well as the encouraging rate at which the field is advancing, we are hopeful that reliable prognostic tau biomarkers that are associated with disease status and progression will be identified and brought into routine clinical practice.

## Figures and Tables

**Table 1:** Keywords used to search for relevant results. *: wildcard operator; ab: terms found in abstract or title; ti: terms found in title; af: terms found in all fields; yr: year of publication.

Ovid: Embase 1996 to 2021 Week 17, Ovid MEDLINE(R) and Epub Ahead of Print, In-Process and Other Non-Indexed Citations, Daily and Versions(R) 1946 to 1 May 2021	
String Number	Term
1	(Alzheimer* AND (plasma OR serum OR blood) AND (biomarker* OR “biological marker”) AND (tau* OR t-tau OR -p-tau* OR ttau OR ptau)).ab.
2	1 NOT (“systematic review” OR review OR summary).ti.
3	Limit 2 to English language
4	Limit 3 to humans
5	Limit 4 to article (Embase only)
6	Limit 5 to yr = “2012 -Current”
7	Remove duplicates from 6

## Data Availability

All data have been made available within the manuscript and Supplementary Tables.

## Supplementary Materials

The following supporting information can be downloaded at: https://www.mdpi.com/article/10.3390/cells12081184/s1, Table S1: Plasma biomarker data extracted from studies included in the data synthesis Table S2: Details of assay and methods employed to measure tau in each study; Table S3: Meta-analysis based on individual technology; Table S4: Quality assessment form; Table S5: Modified QUADAS questions; Figure S1: Equations used to carry out each meta-analysis.

## Abbreviations

Aβ	Amyloid beta
AD	Alzheimer’s disease
ADRDA	Alzheimer’s Disease and Related Disorders Association
AMSTAR	A Measurement Tool to Assess Systematic Reviews
CU	Cognitively unimpaired
DSM	Diagnostic and Statistical Manual of Mental Disorders
ECL	Electrochemiluminescence
ICD	International Classification of Diseases
IMR	Immunomagnetic reduction
LC-MS	Liquid Chromatography–Mass Spectrometry
MCI	Mild cognitive impairment
MSD	Meso Scale Discovery
NIA-AA	National Institute on Aging—Alzheimer’s Association
NINCDS	National Institute of Neurological and Communicative Diseases and Stroke
p-tau	Phosphorylated tau
QUADAS	Quality Assessment of Diagnostic Accuracy Studies
Simoa	Single Molecule Array
STARD	Standards for Reporting Diagnostic Accuracy
t-tau	Total tau

## References

[1] Patterson C. (2018). World Alzheimer Report 2018.

[2] Seppala T.T., Nerg O., Koivisto A.M., Rummukainen J., Puli L., Zetterberg H., Pyykko O.T., Helisalmi S., Alafuzoff I., Hiltunen M. (2012). CSF biomarkers for Alzheimer disease correlate with cortical brain biopsy findings. Neurology.

[3] Skillbäck T., Farahmand B.Y., Rosen C., Mattsson N., Nägga K., Kilander L., Religa D., Wimo A., Winblad B., Schott J.M. (2015). Cerebrospinal fluid tau and amyloid-β1-42 in patients with dementia. Brain.

[4] Morihara T., Kudo T., Ikura Y., Kashiwagi Y., Miyamae Y., Nakamura Y., Tanaka T., Shinozaki K., Nishikawa T., Takeda M. (1998). Increased tau protein level in postmortem cerebrospinal fluid. Psychiatry Clin. Neurosci..

[5] Schöll M., Maass A., Mattsson N., Ashton N.J., Blennow K., Zetterberg H., Jagust W. (2019). Biomarkers for tau pathology. Mol. Cell. Neurosci..

[6] Lewczuk P., Esselmann H., Otto M., Maler J.M., Henkel A.W., Henkel M.K., Eikenberg O., Antz C., Krause W., Reulbach U. (2004). Neurochemical diagnosis of Alzheimer’s dementia by CSF Aβ42, Aβ42/Aβ40 ratio and total tau. Neurobiol. Aging.

[7] Olsson B., Lautner R., Andreasson U., Öhrfelt A., Portelius E., Bjerke M., Hölttä M., Rosén C., Olsson C., Strobel G. (2016). CSF and blood biomarkers for the diagnosis of Alzheimer’s disease: A systematic review and meta-analysis. Lancet Neurol..

[8] Albert M.S., DeKosky S.T., Dickson D., Dubois B., Feldman H.H., Fox N.C., Gamst A., Holtzman D.M., Jagust W.J., Petersen R.C. (2011). The diagnosis of mild cognitive impairment due to Alzheimer’s disease: Recommendations from the National Institute on Aging-Alzheimer’s Association workgroups on diagnostic guidelines for Alzheimer’s disease. Alzheimers Dement..

[9] McKhann G.M., Knopman D.S., Chertkow H., Hyman B.T., Jack C.R., Kawas C.H., Klunk W.E., Koroshetz W.J., Manly J.J., Mayeux R. (2011). The diagnosis of dementia due to Alzheimer’s disease: Recommendations from the National Institute on Aging-Alzheimer’s Association workgroups on diagnostic guidelines for Alzheimer’s disease. Alzheimers Dement..

[10] Sperling R.A., Aisen P.S., Beckett L.A., Bennett D.A., Craft S., Fagan A.M., Iwatsubo T., Jack C.R., Kaye J., Montine T.J. (2011). Toward defining the preclinical stages of Alzheimer’s disease: Recommendations from the National Institute on Aging-Alzheimer’s Association workgroups on diagnostic guidelines for Alzheimer’s disease. Alzheimers Dement..

[11] Barthélemy N.R., Li Y., Joseph-Mathurin N., Gordon B.A., Hassenstab J., Benzinger T., Buckles V., Fagan A.M., Perrin R.J., Goate A.M. (2020). A soluble phosphorylated tau signature links tau, amyloid and the evolution of stages of dominantly inherited Alzheimer’s disease. Nat. Med..

[12] Alzheimer’s Association (2018). 2018 Alzheimer’s disease facts and figures. Alzheimers Dement..

[13] Randall J., Mörtberg E., Provuncher G.K., Fournier D.R., Duffy D.C., Rubertsson S., Blennow K., Zetterberg H., Wilson D.H. (2013). Tau proteins in serum predict neurological outcome after hypoxic brain injury from cardiac arrest: Results of a pilot study. Resuscitation.

[14] Lue L., Pai M., Chen T., Hu C., Huang L., Lin W., Wu C., Jeng J., Blennow K., Sabbagh M.N. (2019). Age-dependent relationship between plasma Aβ40 and Aβ42 and total tau levels in cognitively normal subjects. Front. Aging Neurosci..

[15] Mielke M.M., Hagen C.E., Xu J., Chai X., Vemuri P., Lowe V.J., Airey D.C., Knopman D.S., Roberts R.O., Machulda M.M. (2018). Plasma phospho-tau181 increases with Alzheimer’s disease clinical severity and is associated with tau-and amyloid-positron emission tomography. Alzheimers Dement..

[16] Liu Y., Qing H., Deng Y. (2014). Biomarkers in Alzheimer’s disease analysis by mass spectrometry-based proteomics. Int. J. Mol. Sci..

[17] Jack C.R., Bennett D.A., Blennow K., Carrillo M.C., Dunn B., Haeberlein S.B., Holtzman D.M., Jagust W., Jessen F., Karlawish J. (2018). NIA-AA research framework: Toward a biological definition of Alzheimer’s disease. Alzheimers Dement..

[18] Bossuyt P.M., Reitsma J.B., Bruns D.E., Gatsonis C.A., Glasziou P.P., Irwig L.M., Lijmer J.G., Moher D., Rennie D., De Vet H.C. (2003). Towards complete and accurate reporting of studies of diagnostic accuracy: The STARD initiative. Ann. Intern. Med..

[19] Shea B.J., Grimshaw J.M., Wells G.A., Boers M., Andersson N., Hamel C., Porter A.C., Tugwell P., Moher D., Bouter L.M. (2007). Development of AMSTAR: A measurement tool to assess the methodological quality of systematic reviews. BMC Med. Res. Methodol..

[20] Whiting P., Rutjes A., Dinnes J., Reitsma J., Bossuyt P., Kleijnen J. (2004). Development and validation of methods for assessing the quality of diagnostic accuracy studies. Health Technol. Assess..

[21] Mattsson N., Andreasson U., Persson S., Carrillo M.C., Collins S., Chalbot S., Cutler N., Dufour-Rainfray D., Fagan A.M., Heegaard N.H. (2013). CSF biomarker variability in the Alzheimer’s Association quality control program. Alzheimers Dement..

[22] Friedrich J.O., Adhikari N.K., Beyene J. (2008). The ratio of means method as an alternative to mean differences for analyzing continuous outcome variables in meta-analysis: A simulation study. BMC Med. Res. Methodol..

[23] DerSimonian R., Laird N. (1986). Meta-analysis in clinical trials. Control. Clin. Trials.

[24] Higgins J.P.T., Thompson S.G., Deeks J.J., Altman D.G. (2003). Measuring inconsistency in meta-analyses. BMJ Clin. Res. Ed..

[25] Jack C.R.J., Bennett D.A., Blennow K., Carrillo M.C., Feldman H.H., Frisoni G.B., Hampel H., Jagust W.J., Johnson K.A., Knopman D.S. (2016). A/T/N: An unbiased descriptive classification scheme for Alzheimer disease biomarkers. Neurology.

[26] American Psychiatric Association (2000). Diagnostic and Statistical Manual of Mental Disorders (DSM-IV).

[27] Petersen R.C., Stevens J.C., Ganguli M., Tangalos E.G., Cummings J.L., DeKosky S.T. (2001). Practice parameter: Early detection of dementia: Mild cognitive impairment (an evidence-based review). Report of the Quality Standards Subcommittee of the American Academy of Neurology. Neurology.

[28] McKhann G., Drachman D., Folstein M., Katzman R., Price D., Stadlan E.M. (1984). Clinical diagnosis of Alzheimer’s disease: Report of the NINCDS-ADRDA Work Group under the auspices of Department of Health and Human Services Task Force on Alzheimer’s Disease. Neurology.

[29] Janelidze S., Mattsson N., Palmqvist S., Smith R., Beach T.G., Serrano G.E., Chai X., Proctor N.K., Eichenlaub U., Zetterberg H. (2020). Plasma P-tau181 in Alzheimer’s disease: Relationship to other biomarkers, differential diagnosis, neuropathology and longitudinal progression to Alzheimer’s dementia. Nat. Med..

[30] De Vos A., Jacobs D., Struyfs H., Fransen E., Andersson K., Portelius E., Andreasson U., De Surgeloose D., Hernalsteen D., Sleegers K. (2015). C-terminal neurogranin is increased in cerebrospinal fluid but unchanged in plasma in Alzheimer’s disease. Alzheimers Dement..

[31] Wang T., Xiao S., Liu Y., Lin Z., Su N., Li X., Li G., Zhang M., Fang Y. (2014). The efficacy of plasma biomarkers in early diagnosis of Alzheimer’s disease. Int. J. Geriatr. Psychiatry.

[32] Barthélemy N.R., Bateman R.J., Hirtz C., Marin P., Becher F., Sato C., Gabelle A., Lehmann S. (2020). Cerebrospinal fluid phospho-tau T217 outperforms T181 as a biomarker for the differential diagnosis of Alzheimer’s disease and PET amyloid-positive patient identification. Alzheimers Res. Ther..

[33] Karikari T.K., Pascoal T.A., Ashton N.J., Janelidze S., Benedet A.L., Rodriguez J.L., Chamoun M., Savard M., Kang M.S., Therriault J. (2020). Blood phosphorylated tau 181 as a biomarker for Alzheimer’s disease: A diagnostic performance and prediction modelling study using data from four prospective cohorts. Lancet Neurol..

[34] Thijssen E.H., La Joie R., Strom A., Fonseca C., Iaccarino L., Wolf A., Spina S., Allen I.E., Cobigo Y., Heuer H. (2021). Plasma phosphorylated tau 217 and phosphorylated tau 181 as biomarkers in Alzheimer’s disease and frontotemporal lobar degeneration: A retrospective diagnostic performance study. Lancet Neurol..

[35] Mattsson N., Zetterberg H., Janelidze S., Insel P.S., Andreasson U., Stomrud E., Palmqvist S., Baker D., Tan Hehir C.A., Jeromin A. (2016). Plasma tau in Alzheimer disease. Neurology.

[36] Kukull W.A., Larson E., Reifler B., Lampe T., Yerby M., Hughes J. (1990). The validity of 3 clinical diagnostic criteria for Alzheimer’s disease. Neurology.

[37] Dujardin S., Commins C., Lathuiliere A., Beerepoot P., Fernandes A.R., Kamath T.V., De Los Santos M.B., Klickstein N., Corjuc D.L., Corjuc B.T. (2020). Tau molecular diversity contributes to clinical heterogeneity in Alzheimer’s disease. Nat. Med..

[38] Rózga M., Bittner T., Batrla R., Karl J. (2019). Preanalytical sample handling recommendations for Alzheimer’s disease plasma biomarkers. Alzheimers Dement. Diagn. Assess. Dis. Monit..

[39] Hansson O., Edelmayer R.M., Boxer A.L., Carrillo M.C., Mielke M.M., Rabinovici G.D., Salloway S., Sperling R., Zetterberg H., Teunissen C.E. (2022). The Alzheimer’s Association appropriate use recommendations for blood biomarkers in Alzheimer’s disease. Alzheimers Dement..

[40] Wang Y., Mandelkow E. (2016). Tau in physiology and pathology. Nat. Rev. Neurosci..

[41] Blennow K. (2021). Plasma pTau isoform assessment across disease stages. Alzheimers Dement..

[42] Lewczuk P., Matzen A., Blennow K., Parnetti L., Molinuevo J.L., Eusebi P., Kornhuber J., Morris J.C., Fagan A.M. (2017). Cerebrospinal fluid Aβ 42/40 corresponds better than Aβ 42 to amyloid PET in Alzheimer’s Disease. J. Alzheimers Dis..

[43] Chen Z., Mengel D., Keshavan A., Rissman R.A., Billinton A., Perkinton M., Percival-Alwyn J., Schultz A., Properzi M., Johnson K. (2019). Learnings about the complexity of extracellular tau aid development of a blood-based screen for Alzheimer’s disease. Alzheimers Dement..

[44] Neddens J., Temmel M., Flunkert S., Kerschbaumer B., Hoeller C., Loeffler T., Niederkofler V., Daum G., Attems J., Hutter-Paier B. (2018). Phosphorylation of different tau sites during progression of Alzheimer’s disease. Acta Neuropathol. Commun..

[45] Ashton N.J., Pascoal T.A., Karikari T.K., Benedet A.L., Lantero-Rodriguez J., Brinkmalm G., Snellman A., Schöll M., Troakes C., Hye A. (2021). Plasma p-tau231: A new biomarker for incipient Alzheimer’s disease pathology. Acta Neuropathol..

[46] Hampel H., Frank R., Broich K., Teipel S.J., Katz R.G., Hardy J., Herholz K., Bokde A.L., Jessen F., Hoessler Y.C. (2010). Biomarkers for Alzheimer’s disease: Academic, industry and regulatory perspectives. Nat. Rev. Drug Discov..

[47] Arastoo M., Lofthouse R., Penny L.K., Harrington C.R., Porter A., Wischik C.M., Palliyil S. (2020). Current progress and future directions for tau-based fluid biomarker diagnostics in Alzheimer’s disease. Int. J. Mol. Sci..

