# Supplementary material for: Utility of Blood-Based Tau Biomarkers for Mild Cognitive Impairment and Alzheimer’s Disease: Systematic Review and Meta-Analysis
Source: Cells. 2023 Apr 18;12(8):1184. doi: 10.3390/cells12081184 (PMC10136726; doi:10.3390/cells12081184)
Supplement: Supplementary file 1 [file cells-12-01184-s001.zip › cells-2234756-supplementary.pdf]

Supplementary Tables and figures

Study (cohort)	Type of tau	AD* (n)	MCI* (n)	CU (n)	Technology used
Chen 2020	p-tau181	37.94±14.04 (18)	23.19±9.02 (23)	32.4± 10.39 (24)	ELISA
Jiao 2015	p-tau181	150±57.7 (156)	nd	107±57.8 (129)	ELISA
Tsai 2020	p-tau181	3.8± 0.7 (19)	3.7± 0.73 (24)	3.4± 0.63 (10)	IMR
Hasegawa 2020	p-tau181	9.3±3.24 (12)	5.33± 1.59 (12)	4.49±2.12 (9)	IMR
Tsai 2019	p-tau181	4.09±0.94 (37)	3.82±0.71 (40)	3.53±0.55 (13)	IMR
Yang 2018	p-tau181	6.14±1.59 (21)	4.41±1.85 (29)	2.46±1.09 (23)	IMR
Barthelemy 2020	p-tau181	nd	2.9± 1 (24)	2.1±0.7 (31)	LC-MS
Zettergren 2021	p-tau181	23.6±8.2 (148)	18.3±11.0 (434)	14.9±8.7 (236)	SIMOA
Karikari 2021	p-tau181	25.5±8.6 (137)	22.8±9.9(209)	14.2±9 (268)	SIMOA
Simren 2021	p-tau181	19.43±7.57 (103)	13.13±6.21 (107)	8.85± 4.45 (99)	SIMOA
Ashton 2021	p-tau181	25.21±7.8 (42)	16.26±6.7 (54)	10.91± 3.3 (159)	SIMOA
Brickman (pathological) 2021	p-tau181	1.93± 1.14 (33)	nd	1.06±0.81 (80)	SIMOA
Brickman (clinical) 2021	p-tau181	1.24±1.09 (131)	nd	0.86±0.73 (169)	SIMOA
Karikari (BioFINDER-2) 2020	p-tau181	19.2±9.4 (126)	12.5±8.6 (191)	9.4±6 (337)	SIMOA
Karikari (TRIAD) 2020	p-tau181	25±7.8 (33)	15.6±5.6 (28)	9.3±2.9 (91)	SIMOA
O'Connor 2020	p-tau181	23.7±10.5 (19)	nd	9.7±9.3 (27)	SIMOA
Benussi 2020	p-tau181	16.4± 8.7(63)	nd	5.4± 1(63)	SIMOA
Lantero 2020	p-tau181	28.4±9.6 (77)	nd	9.3± 9.4 (28)	SIMOA
Palmqvist 2020	p-tau181	2.87± 4.28 (34)	nd	2.52± 2.41 (47)	SIMOA
Mielke 2018	p-tau181	11.6±4.1 (40)	9±13.9 (57)	6.4±6.4 (172)	SIMOA
Tatebe 2017	p-tau181	0.171±0.166 (20)	nd	0.0405±0.0756 (15)	SIMOA

Barthelemy 2020	p-tau217	nd	0.31±0.16 (24)	0.07±0.03 (31)	LC-MS
Palmqvist 2020	p-tau217	7.75±5.34 (34)	nd	1.79±1.95 (47)	MSD-ECL
Mattsson 2020	p-tau217	nd	4.16±3.11 (49)	1.18±1.64 (88)	MSD-ECL
Brickman (pathological) 2021	p-tau217	0.51±0.4 (33)	nd	0.19± 0.16 (80)	SIMOA
Brickman (clinical) 2021	p-tau217	0.32±0.32 (131)	nd	0.18±0.17 (169)	SIMOA
Ashton 2021	p-tau231	29.22±8.2 (42)	19.45±7.1 (54)	14.94±4.1 (159)	SIMOA
Palmqvist (BioFINDER) 2019	t-tau	16.7±6 (64)	19.1±5.2 (265)	16.6±4.7 (513)	Elecsys-ECL
Palmqvist (Germany) 2019	t-tau	15.3±4.5 (94)	14.2±4.7 (109)	13.8±4 (34)	Elecsys-ECL
Nam 2020	t-tau	351.9±223.8 (20)	263± 37.12 (30)	245.6±33.76 (26)	ELISA
Chen 2020	t-tau	303.85±257.5 (18)	90.56±26.92 (23)	132.47± 30.01 (24)	ELISA
Neergaard (tau-A) 2018	t-tau	25.8±19.7 (232)	nd	26.8±14.5 (4771)	ELISA
Neergaard (tau-C) 2018	t-tau	19.7±12.7 (232)	nd	20.3±10.4 (4771)	ELISA
Rani 2017	t-tau	451.76±240.8 2 (45)	nd	836.93± 369.31 (45)	ELISA
Jiao 2015	t-tau	227.1±102.2 (156)	nd	181±103.2 (129)	ELISA
de Vos 2015	t-tau	40.2±63.1 (20)	17.7 (10.3, 26.2) (20) †	24.6±33.6 (29)	ELISA
Wang 2014	t-tau	213.95±44.57 (97)	209.61±39.65 (51)	214.94±43.23 (122)	ELISA
Sparks 2012	t-tau	530±193.6 (49)	729.8±225.6 (47)	819.5±294.4 (110)	ELISA
Chiu (validation) 2020	t-tau	40.9±14.4 (73)	30.5±11.1 (33)	20.2±10.5 (134)	IMR
Chiu (derivation) 2020	t-tau	47.47±19.1 (59)	33.33±7.7 (34)	13.83±6.5 (67)	IMR
Jiao 2020	t-tau	25.91± 8.12 (40)	nd	20.65±3.52 (57)	IMR
Tsai 2020	t-tau	26.4± 4.2 (19)	23.7± 3.7 (24)	23± 3.5 (10)	IMR

Hasegawa 2020	t-tau	20.3±7.04 (12)	13± 3.05 (12)	12± 4.59 (9)	IMR
Tsai 2019	t-tau	27.1±4.8 (37)	24.5±4 (40)	22.5±3.4 (13)	IMR
Fan 2018	t-tau	39.4±5.8 (16)	29.7±8.7 (25)	14.3±6.4 (39)	IMR
Yang 2018	t-tau	37.54±12.29 (21)	32.98±10.18 (29)	18.85±10.16 (23)	IMR
Yang 2017	t-tau	72.2±9.9 (29)	33.33±7.77 (24)	13.37±7.77 (66)	IMR
Lue (USA) 2017	t-tau	34.52±15 (16)	nd	20.48±4.96 (16)	IMR
Lue (Taiwan) 2017	t-tau	52.47±15.14 (31)	nd	13.98±14.76 (61)	IMR
Lee 2017	t-tau	47.5±18.9 (62)	nd	15± 7.3 (34)	IMR
Chiu 2014	t-tau	53.9±11.7 (10)	32.7±5.8 (20)	15.6±6.9 (30)	IMR
Tzen 2014	t-tau	46.7±2(14)	33.5±2.2 (11)	13.5±5.5 (20)	IMR
Chiu 2013	t-tau	73.01±139.38 (30)	55.57±22.87 (31)	16.16±9.09 (107)	IMR
Barthelemy 2020	t-tau	nd	22.7±6.9 (24)	20.4±6.3 (31)	LC-MS
Palmkvist 2020	t-tau	3.74± 2.23 (34)	nd	2.45±1.81 (47)	SIMOA
Hsu 2021	t-tau	2.80±1.50 (55)	nd	1.80±0.9 (22)	SIMOA
Simren 2021	t-tau	3.21±2.48 (103)	2.69±1.08 (107)	2.36± 1.07 (99)	SIMOA
Deniz 2021	t-tau	3.75±2.36 (159)	nd	3.84± 2.26 (162)	SIMOA
Brickman (pathological) 2021	t-tau	4.12±2.37 (33)	nd	4.3±3.22 (80)	SIMOA
Brickman (clinical) 2021	t-tau	4.9± 2.06 (131)	nd	4.94±2.13 (169)	SIMOA
Startin 2020	t-tau	2.272±4.542 (27)	nd	2.38± 2.526 (27)	SIMOA
Fossati 2020	t-tau	3.67±1.06 (29)	nd	2.74± 0.76 (68)	SIMOA
Pase 2019	t-tau	4.91±1.69 (132)	nd	4.36±2.19 (1321)	SIMOA

Li 2019	t-tau	5.47±2.69 (53)	6.68±4.03 (22)	4.62±0.5 (9)	SIMOA
Chen (discovery - FL) 2019	t-tau	3.13±5.95 (25)	2.96±4.52 (20)	2.75±5.32 (19)	SIMOA
Chen (validation - NT1) 2019	t-tau	3.4±0.29 (23)	3.42±0.23 (22)	2.36±0.17 (41)	SIMOA
Mielke 2018	t-tau	7.2±2.8 (40)	5.9±2.8 (57)	5.9±1.9 (172)	SIMOA
Mattsson (ADNI) 2016	t-tau	3.13±1.50 (179)	2.71±1.32 (195)	2.58±1.19 (189)	SIMOA
Zetterberg 2013	t-tau	8.8±10.1 (54)	4.68±4.25 (75)	4.43±2.83 (25)	SIMOA

Table S1. Plasma biomarker data extracted from studies included in the data synthesis. Studies are identified by the first author's name, the name of the cohort within the study, if applicable, and ordered chronologically by year of publication. *Data presented as mean±SD pg/ml unless specified otherwise. †Data provided as median (IQR). n: number of patients. AD: Alzheimer's disease; MCI: mild cognitive impairment; CU: cognitively unimpaired; nd: No data; SD: standard deviation;. IQR: interquartile range; IMR: immunomagnetic reduction;. ELISA: enzyme-linked immunosorbent assay; SIMOA: single molecule array; LC-MS: Liquid chromatography-mass spectrometry; MSD: Meso Scale Discovery.

Assay type	Study	Tau type	Assay details
Elecsys-ECL	Palmqvist *et al.*, 2019	t-tau	Elecsys immunoassay (Roche diagnostics). Measured on Cobas e 601 analyzer (Roche Diagnostics)
MSD-ECL	Mattsson-Carlgren et al., 2020	p-tau217	Biotinylated-IBA493 was used as a capture antibody (anti-P-tau217) and SULFO-TAG-4G10-E2 (anti-Tau) for the detector
SIMOA	Palmqvist et al., 2020	t-tau	SIMOA tau 2.0 kit (Quanterix). Measured on a SIMOA HD-1 (Quanterix)
MSD-ECL		p-tau181	Biotinylated-AT270 MAb (Thermo Fisher Scientific Inc.) for capture. SULFO-TAG-LRL (anti-tau mAb; Lilly Research Laboratory) for detection SIMOA
MSD-ECL		p-tau217	Biotinylated-IBA493 was used as a capture antibody and SULFO-TAG-4G10-E2 (anti-Tau) as the detector
MSD-ECL	Mielke et al., 2018	p-tau181	Biotinylated-AT270 mAb (Thermo Fisher Scientific Inc.) for capture. MSULFO-TAG-LRL (Lilly Research Laboratory) for detection
SIMOA		t-tau	SIMOA Tau 2.0 kit (Quanterix). Measured on SIMOA HD-1 (Quanterix)
MSD-ECL	Brickman et al., 2021	p-tau217	IBA493 mAb for capture. SULFO-TAG-Ru-4G10-E2 for detector
MSD-ECL	Brickman et al., 2021	p-tau181	AT270 mAb for capture. SULFO-TAG-Ru-4G10-E2 for detector
SIMOA		t-tau	Human Neurology 3-Plex A assay kit (Quanterix). Measured on a SIMOA HD-1 (Quanterix)
SIMOA	Karikari et al., 2020	p-tau181	AT270 mouse mAb coupled to paramagnetic beads. Tau12 antibody (Merck) for detection. Measured on SIMOA HD-1 (Quanterix)
SIMOA	de Wolf et al., 2020	t-tau	Human Neurology 3-Plex A assay (N3PA). Measured on SIMOA HD-1 (Quanterix)
SIMOA	Pase et al., 2019	t-tau	SIMOA tau 2.0 kit (Quanterix). Measured on SIMOA HD-1 (Quanterix)
SIMOA	Li et al., 2019	t-tau	Human Neurology 3-Plex A assay kit (Quanterix). Measured on a SIMOA HD-1 (Quanterix)
SIMOA	Chen et al., 2019	t-tau	Full-length: TauAB (aa 425-441) for capture; NT1 region: BT2 for capture; NT2 region: ADx202 (aa 218-224) for capture. Tau12 (Merck) for detection
SIMOA	Tatebe et al., 2017	p-tau181	Modified Human Total Tau kit (SIMOA Tau 2.0, Quanterix). AT270 mAb (Thermo Fisher Scientific) for detection
SIMOA	Zetterberg et al., 2013	t-tau	Tau-5 mAb (Covance) for capture, HT7 and BT2 mAb (Pierce/Thermo Fisher Scientific) for detection
SIMOA	Park et al., 2019	t-tau	SIMOA tau 2.0 kit (Quanterix)

Assay type	Study	Tau type	Assay details
		p-tau181	Phospho-tau thr181 immunoassay (Quanterix)
SIMOA	Mattsson et al., 2016	t-tau	Human total tau kit (Quanterix)
SIMOA	Startin et al., 2019	t-tau	Human total tau kit (Quanterix)
SIMOA	Fossati et al., 2019	t-tau	SIMOA assay using a capture antibody which recognizes amino acid 16-24 and a detector antibody recognizing amino acid 218-222
SIMOA	Smiren et al., 2020	t-tau	Advantage Neuro 3-plex (Quanterix). Measured on a SIMOA HD-1 (Quanterix)
		p-tau181	AT270 mouse mAb coupled to paramagnetic beads. Tau12 antibody used for detection. Measured on SIMOA HD-1 (Quanterix)
SIMOA	Lantero et al., 2020	p-tau181	AT270 mouse mAb coupled to paramagnetic beads. Tau12 antibody used for detection
SIMOA	Deniz et al. 2021	t-tau	Human total tau kit (Quanterix). Analysed on SIMOA HD1-Analyzer (Quanterix, Lexington, MA)
SIMOA	Ashton et al., 2021	p-tau181	In-house SIMOA assay. Measured on HD-X instrument (Quanterix)
SIMOA	Karikari et al., 2021	p-tau181	AT270 mouse mAb coupled to paramagnetic beads. Tau12 antibody used for detection
SIMOA	O'connor et al., 2021	p-tau181	AT270 mouse mAb coupled to paramagnetic beads. N-terminal antibody used for detection
SIMOA	Zettergren et al., 2021	p-tau181	AT270 mouse mAb coupled to paramagnetic beads. Tau12 antibody used for detection
ELISA	Neergaard et al., 2018	t-tau	Solid phase competitive ELISA. MAbs detect an ADAM10-generated cleavage site at Ala152 (Tau-A) and the caspase-3-generated cleavage site at Asp421 (Tau-C)
ELISA	Sparks et al., 2012	t-tau	ELISA. KHB0042 (Invitrogen)
ELISA	Wang et al., 2014	t-tau	ELISA. KHB00412 (Invitrogen)
		p-tau181	ELISA. KHO0631 (Invitrogen)
ELISA	Jiao et al., 2015	t-tau	ELISA. KUB0041 (Invitrogen)
		p-tau181	ELISA. KUO0631 (Invitrogen)
ELISA	De Vos et al., 2015	t-tau	77E9 mAb for capture. ADx215 for detection
ELISA	Rani et al. 2017	t-tau	Cusabio tau kit (CSB-E12011h)
ELISA	Nam et al., 2020	t-tau	MyBioSource (MBS022635)
ELISA (chemiluminescence)	Hasegawa et al. 2020	t-tau	MAb recognising aa16-24 for capture. Mab recognising aa218-225 for detection
		p-tau181	MAb recognising p-tau181 for capture. MAb recognising aa16-24 for detection
IMR	Chiu et al., 2020	t-tau	T9450 (Sigma) immobilised antibody assay. Measured on a magnetosusceptometer (XacPro-S, MagQu)
IMR	Chiu et al., 2013	t-tau	T9450 (Sigma) immobilised antibody assay. Measured on a magnetosusceptometer (XacPro-S, MagQu)
IMR	Chiu et al., 2014	t-tau	Dextran coated Fe3O4 magnetic nanoparticles (MF-DEX-0060, MagQu) functionalised against tau with tau-441 (Sigma). Measured on a magnetosusceptometer (XacPro-S, MagQu)

Assay type	Study	Tau type	Assay details
IMR	Yang et al., 2018	p-tau181	Dextran coated Fe3O4 magnetic nanoparticles (MF-DEX-0060, MagQu) functionalised against p-tau181 MN1050 (Thermo Fisher Scientific Inc.)
		t-tau	Dextran coated nanoparticles functionalized with MF-TAU-0060 (MagQu)
IMR	Fan et al., 2018	t-tau	Tau46 (T9450, Sigma) conjugated to dextran-coated magnetic nanoparticles. Analysed on IMR analyser (XacPro-S, MagQu)
IMR	Lue et al., 2017	t-tau	Dextran coated nanoparticles functionalized with MF-TAU-0060 (MagQu) and measured on a magnetosusceptometer (XacPro-S, MagQu)
		p-tau181	
IMR	Tsai et al., 2019/ 2020	t-tau	Dextran coated nanoparticles functionalized with MF-TAU-0060 (MagQu) and measured on a magnetosusceptometer (XacPro-S, MagQu)
		p-tau181	Dextran coated nanoparticles functionalized with MF-PT1-0060 (MagQu) and measured on a magnetosusceptometer (XacPro-S, MagQu)
IMR	Jiao et al. 2020	t-tau	Dextran coated nanoparticles functionalized with MF-TAU-0060 (MagQu)
LC-MS	Barthelemy et al. (date?)	p-tau 217	nanoAcquity ultra performance liquid chromatography system (Waters) coupled to an Orbitrap Tribrid Eclipse mass spectrometer (Thermo Fisher Scientific)
		p-tau181	
		t-tau	

Table S2. Details of assay and methods employed to measure tau in each study. The studies have been grouped by broad categories according to the type of assay used, and these categories have been colour coded. Details of the assays included, where applicable: name of the antibody or reagent, manufacturer, and method of measurement. mAb: monoclonal antibody. SIMOA: single molecule assay. ELISA: enzyme-linked immunosorbent assay. IMR: immunomagnetic reduction. MSD: Meso scale discovery. LC-MS: Liquid chromatography–mass spectrometry.

A

AD	Assay	Effect size	Variance	Standard error	Upper CI	Lower CI	I^2	n
p-tau181	Simoa	2.09	0.000	0.020	2.13	2.05	99.90	13
	ELISA	1.35	0.003	0.050	1.45	1.25	99.86	2
	IMR	1.38	0.002	0.040	1.46	1.30	99.77	4
t-tau	Simoa	1.24	0.026	0.162	1.56	0.92	99.07	14
	ELISA	1.16	0.034	0.184	1.53	0.80	99.66	10
	IMR	2.29	0.000	0.021	2.33	2.25	99.91	15
	Electrochemiluminescence	1.05	0.001	0.036	1.12	0.97	99.88	2

B

MCI	Assay	Effect size	Variance	Standard error	Upper CI	Lower CI	I^2	n
p-tau181	Simoa	1.46	0.037	0.193	1.84	1.08	95.30	7
	IMR	1.28	0.066	0.257	1.79	0.78	91.16	4
t-tau	Simoa	1.11	0.002	0.041	1.19	1.03	99.33	6
	ELISA	0.99	0.000	0.020	1.02	0.95	99.84	5
	IMR	1.95	0.024	0.154	2.25	1.65	99.33	11
	Electrochemiluminescence	1.09	0.127	0.356	1.79	0.39	96.33	2

Table S3. Meta-analysis based on individual technology for both t-tau and p-tau181 in AD compared with CU (A) and MCI compared with CU (B). IMR: Immunomagnetic resonance, CI: Confidence interval, I^2: a measure of inconsistency which represents the percentage of the chi-squared statistic not explained by the variation within the studies.

Quality Assessment

Study	1	2	3	4	5	6	7	8	9	10	11	12	13	14	15	16	17	18	Control
Karikari *et al.,* (2020)	Y	Y	Y	Y	Y	Y	Y	Y	Y	U	U	Y	NA	NA	Y	Y	Y	Y	Age-matched controls
Chiu *et al.,* (2020)	Y	Y	Y	Y	Y	Y	Y	Y	Y	U	U	Y	NA	NA	Y	Y	N	Y	Age-matched controls
de Wolf *et al.,* (2020)	Y	Y	Y	Y	Y	Y	Y	Y	Y	U	U	Y	NA	NA	N	Y	Y	Y	Age-matched controls
Janelidze *et al.,* (2020)	Y	N	Y	Y	Y	Y	Y	Y	Y	U	U	Y	NA	NA	N	Y	Y	Y	Age-matched controls and post-mostem
Palmqvist *et al.,* (2019)	Y	Y	Y	Y	Y	Y	Y	Y	Y	U	U	Y	NA	NA	Y	Y	Y	Y	Age-matched controls
Pase *et al.,* (2019)	Y	Y	Y	Y	Y	Y	Y	Y	Y	U	U	Y	NA	NA	N	Y	N	Y	Age-matched controls
Li *et al.,* (2019)	Y	Y	Y	Y	Y	Y	Y	Y	Y	U	U	Y	NA	NA	N	Y	N	Y	Age-matched controls
Chen *et al.,* (2019)	Y	Y	Y	Y	Y	Y	Y	Y	Y	U	U	Y	NA	NA	N	Y	Y	Y	Age-matched controls
Neergaard *et al.,* (2018)	Y	N	Y	Y	Y	Y	Y	Y	Y	U	U	Y	NA	NA	N	Y	N	Y	Age-matched controls (post-menopausal women)
Tatebe *et al.,* (2017)	Y	Y	Y	Y	Y	Y	Y	Y	Y	U	U	Y	NA	NA	N	Y	Y	Y	Age-matched controls
Mattsson *et al.,* (2016)	Y	Y	Y	Y	Y	Y	Y	Y	Y	U	U	Y	NA	NA	N	Y	Y	Y	Age-matched controls
Sparks *et al.,* (2012)	Y	N	Y	Y	Y	Y	Y	Y	Y	U	U	Y	NA	NA	N	N	N	N	Age-matched controls
Chiu *et al* ., (2013)	Y	N	Y	Y	Y	Y	Y	Y	Y	U	U	Y	NA	NA	N	N	Y	Y	Age-matched controls
Zetterberg *et al.,* (2013)	Y	N	Y	Y	Y	Y	Y	Y	Y	U	U	Y	NA	NA	N	Y	N	Y	Age-matched controls
Wang *et al.,* (2014)	Y	Y	Y	Y	Y	Y	Y	Y	Y	U	U	Y	NA	NA	N	N	Some	Y	Age-matched controls
Chiu *et al* ., (2014)	Y	Y	Y	Y	Y	Y	Y	Y	Y	U	U	Y	NA	NA	N	N	Y	N	Age-matched controls
Mielke *et al.,* (2018)	Y	N	Y	Y	Y	Y	Y	Y	Y	U	U	Y	NA	NA	Y	Y	N	Y	Age-matched controls
Yanga *et al.,* (2018)	Y	Y	Y	Y	Y	Y	Y	Y	Y	U	U	Y	NA	NA	N	Y	Y	Y	Age-matched controls
Park *et al.,* (2019)	Y	Y	Y	Y	Y	Y	Y	Y	Y	U	U	Y	NA	NA	N	Y	N	Y	Age-matched controls
Jiao *et al.,* (2015)	Y	Y	Y	Y	Y	Y	Y	Y	Y	U	U	Y	NA	NA	Y	Y	Y	Y	Age-matched controls
de Vos *et al.,* (2015)	Y	Y	Y	Y	Y	Y	Y	Y	Y	U	U	Y	NA	NA	N	Y	Y	Y	Age-matched controls
Park *et al.,* (2019)	Y	Y	Y	Y	Y	Y	Y	Y	Y	U	U	Y	NA	NA	N	Y	N	Y	Age-matched controls
Nam *et al.,* (2020)	Y	Y	Y	Y	Y	Y	Y	Y	Y	U	U	Y	NA	NA	N	N	Y	Y	Age-matched controls
Chen *et al.,* (2020)	N	Y	Y	Y	Y	Y	Y	Y	Y	U	U	Y	NA	NA	N	N	N	Y	Age-matched controls

Study	1	2	3	4	5	6	7	8	9	10	11	12	13	14	15	16	17	18	Control
Rani *et al.,* (2017)	Y	Y	Y	Y	Y	Y	Y	Y	Y	U	U	Y	NA	NA	N	N	Y	Y	Age-matched controls
Jiao et al., (2020)	Y	Y	Y	Y	Y	Y	Y	Y	Y	U	U	Y	NA	NA	N	N	Y	Y	Age-matched controls
Tsai *et al.,* (2020)	Y	Y	Y	Y	Y	Y	Y	Y	Y	U	U	Y	NA	NA	N	Y	Y	Y	Age-matched controls
Hasegawa *et al.,* (2020)	Y	Y	Y	Y	Y	Y	Y	Y	Y	U	U	Y	NA	NA	N	N	Y	Y	Age-matched controls
Tsai *et al.,* (2019)	Y	Y	Y	Y	Y	Y	Y	Y	Y	U	U	Y	NA	NA	N	Y	N	Y	Age-matched controls
Fan *et al.,* (2018)	Y	Y	Y	Y	Y	Y	Y	Y	Y	U	U	Y	NA	NA	N	Y	Y	Y	Age-matched controls
Yang *et al.,* (2017)	Y	Y	Y	Y	Y	Y	Y	Y	Y	U	U	Y	NA	NA	N	Y	Y	Y	Age-matched controls
Lue *et al.,* (2017)	Y	Y	Y	Y	Y	Y	Y	Y	Y	U	U	Y	NA	NA	N	Y	Y	Y	Age-matched controls
Lee *et al.,* (2017)	Y	Y	Y	Y	Y	Y	Y	Y	Y	U	U	Y	NA	NA	N	N	N	Y	Age-matched controls
Barthélemy *et al.,* (2020)	Y	Y	Y	Y	Y	Y	Y	Y	Y	U	U	Y	NA	NA	N	Y	Y	N	Age-matched controls
Tzen *et al.,* (2014)	Y	Y	Y	Y	Y	Y	Y	Y	Y	U	U	Y	NA	NA	N	N	N	Y	Age-matched controls
Palmqvist *et al* ., (2020)	Y	Y	Y	Y	Y	Y	Y	Y	Y	U	U	Y	NA	NA	N	N	Y	Y	Age-matched controls
Mattsson-Carlgren *et al.,* (2020)	Y	Y	Y	Y	Y	Y	Y	Y	Y	U	U	Y	NA	Y	N	N	Y	Y	Age-matched controls
Zettergren *et al.,* (2021)	Y	N	Y	Y	Y	Y	Y	Y	Y	U	U	Y	NA	NA	N	N	N	Y	Age-matched controls
Hsu *et al.,* (2021)	Y	Y	Y	Y	Y	Y	Y	Y	Y	U	U	Y	NA	NA	Y	Y	N	Y	Age-matched controls
Karikari *et al.,* (2021)	Y	Y	Y	Y	Y	Y	Y	Y	Y	U	U	Y	NA	NA	N	N	Y	Y	Age-matched controls
Simren *et al.,* (2021)	Y	Y	Y	Y	Y	Y	Y	Y	Y	U	U	Y	NA	NA	N	Y	Y	Y	Age-matched controls
Deniz *et al.,* (2021)	N	Y	Y	Y	Y	Y	Y	Y	Y	U	U	Y	NA	NA	N	Y	N	Y	Age-matched controls
Ashton *et al.,* (2021)	Y	Y	Y	Y	Y	Y	Y	Y	Y	U	U	Y	NA	NA	N	Y	Y	Y	Age-matched controls
Brickman *et al.,* (2021)	Y	Y	Y	Y	Y	Y	Y	Y	Y	U	U	Y	NA	NA	N	N	Y	Y	Age-matched controls
O'Connor *et al.,* (2020)	Y	Y	Y	Y	Y	Y	Y	Y	Y	U	U	Y	NA	NA	N	Y	Y	Y	Age-matched controls
Benussi *et al.,* (2020)	Y	Y	Y	Y	Y	Y	Y	Y	Y	U	U	Y	NA	NA	N	Y	Y	Y	Age-matched controls
Lantero Rodriguez *et al.,* (2020)	Y	Y	Y	Y	Y	Y	Y	Y	Y	U	U	Y	NA	NA	N	Y	Y	Y	Age-matched controls
Startin *et al.,* (2019)	Y	Y	Y	Y	Y	Y	Y	Y	Y	U	U	Y	NA	NA	N	N	N	Y	Age-matched controls
Fossati *et al.,* (2020)	Y	Y	Y	Y	Y	Y	Y	Y	Y	U	U	Y	NA	NA	N	Y	Y	Y	Age-matched controls

Table S4. Quality assessment form. Questions were obtained from QUADAS, AMSTAR and STARD.

QUADAS modified

1	Was the spectrum of participants representative of the patients who will receive the test in practice? Yes No Unclear N/A
2	Were selection criteria clearly described? Yes No Unclear N/A
3	Was the reference standard likely to classify the target condition correctly? Yes No Unclear N/A
4	Was the period between performance of the reference standard and the index test short enough to be reasonably sure that the target condition did not change between the two tests? Yes No Unclear N/A
5	Did the whole sample or a random selection of the sample receive verification using the reference standard? Yes No Unclear N/A
6	Did participants receive the same reference standard regardless of the index test result? Yes No Unclear N/A
7	Was the reference standard independent of the index test? (that is, the index test did not form part of the reference standard)? Yes No Unclear N/A
8	Was the execution of the index test described in sufficient detail to permit its replication? Yes No Unclear N/A
9	Was the execution of the reference standard described in sufficient detail to permit its replication? Yes No Unclear N/A
10	Were the index test results interpreted without knowledge of the results of the reference standard? Yes No Unclear N/A
11	Were the reference standard results interpreted without knowledge of the results of the index test? Yes No Unclear N/A
12	Were the same clinical data available when the test results were interpreted as would be available when the test is used in practice? Yes No Unclear N/A
13	Were uninterpretable, indeterminate or intermediate test results reported? Yes No Unclear N/A
14	Were withdrawals from the study explained? Yes No Unclear N/A
15	Were the number, training, and expertise of the persons executing and reading the index tests and the reference standard described? Yes No Unclear N/A
16	Were methods for calculating test reproducibility described? Yes No Unclear N/A
17	Were estimates of diagnostic accuracy and measures of statistical uncertainty (e.g., 95% confidence intervals) reported? Yes No Unclear N/A
18	Was the conflict of interest stated? Yes No Unclear N/A

Table S5. Modified QUADAS questions.

A

$$95\%CI = \exp\left\{\left[\ln\left(\frac{mean_{exp}}{mean_{contr}}\right)\right] \pm 1.96\sqrt{\frac{1}{n_{exp}}\left(\frac{sd_{exp}}{mean_{exp}}\right)^2 + \frac{1}{n_{contr}}\left(\frac{sd_{contr}}{mean_{contr}}\right)^2}\right\}$$

B

1

$$\Theta_{IV(FE)} = \frac{\Sigma_{i=1,k} w_i \times \Theta_i}{\Sigma_{i=1,k} w_i}$$

2

$$Q = \Sigma_{i=1,k}\, w_i \times (\Theta_i - \Theta_{IV(FE)})^2$$

which has a χ^2 distribution with k-1 degrees of freedom when $\tau^2 = 0$. An estimate of τ^2 follows:

3a

$$t^2 = \frac{Q-(k-1)}{\Sigma_{i=1,k} w_i - \frac{\Sigma_{i=1,k}(w_i)^2}{\Sigma_{i=1,k} w_i}} \quad \text{if } Q \geq k-1, \text{ and}$$

3b

$$t^2 = 0 \quad \text{if } Q < k-1$$

4

$$w_i^* = 1/(w_i^{-1} + \tau^2)$$

5

$$\Theta_{IV(RE)} = \frac{\Sigma_{i=1,k} w_i^* \times \Theta_i}{\Sigma_{i=1,k} w_i^*} \quad \text{with variance } (\Theta_{IV(RE)}) = 1 / \Sigma_{i=1,k} w_i^*$$

Figure S1. Equations used to carry out each meta-analysis. **A**. The 95% CI was calculated taking the exponent (exp) of the natural log (ln) or the ratio of the means of experiment (exp, disease cohort) over control (contr) plus or minus the square root of the variance. **B**. Random effects model to calculate overall effect size from studies pooled in each meta-analysis. The numbers on the margin provide guidance on the steps involved. FE: fixed effects. RE: random effects. IV: inverse variance. i: study number (1 to k). k: number of trials in each meta-analysis. n: number of participants. Q: Cochran's Q statistic for heterogeneity. sd: standard deviation. wi: weighting of study i. wi*: weighting of study i incorporating the variance due to heterogeneity. θ: effect size. Figure adapted from Friedrich et al., (2008).
